# The concept, intention, and evaluation of the term *treatment-refractory* meningioma

**DOI:** 10.1007/s11060-025-05154-2

**Published:** 2025-08-04

**Authors:** Lasse Rehné Jensen, Andrea Daniela Maier, Tareq A. Juratli, Stéphane Goutagny, Luca Bertero, Thomas Graillon, Benjamin Brokinkel, Tejpal Gupta, Sverre Helge Torp, Roberta Rudà, Paul M. Clement, Martijn van Essen, María Dolores Tabernero, Konstantinos Gousias, Álvaro Otero Rodríguez, Jong Hee Chang, Chang-Ok Suh, Andrés Felipe Cardona, Oscar Arrieta, Alejandro Ruiz-Patiño, Daniela A. Bota, Maya Hrachova, David Scheie, Bjarne Winther Kristensen, Tina Nørgaard Munch, Ian Law, Kåre Fugleholm, Torstein Ragnar Meling, Julia Furtner, Matthias Preusser, Martin Alexander Walter, Tiit Mathiesen, Christian Mirian

**Affiliations:** 1 Department of Neurosurgery, Copenhagen University Hospital, Blegdamsvej 9, Copenhagen, 2100, +4524262220 Denmark; 2 Department of Pathology, Bartholin Institute, Rigshospitalet, Copenhagen University Hospital, Copenhagen, Denmark; 3 Department of Neurosurgery, Faculty of Medicine, University Hospital Carl Gustav Carus, Technische Universität Dresden, Dresden, Germany; 4 Université Paris Cité, Inserm UMRS 1144, Paris, France; 5 Assistance Publique Hôpitaux de Paris, Hôpital Beaujon, neurosurgery, Clichy, France; 6 Pathology Unit, Department of Medical Sciences, University of Turin, Turin, Italy; 7 Pathology Unit, Department of Medical Sciences, University and Città della Salute e della Scienza University Hospital of Turin, Turin, Italy; 8 Department of Neurosurgery, Aix Marseille Univ, APHM, INSERM, MMG, Hospital La Timone, Marseille, France; 9 Department of Neurosurgery, University of Münster, Münster, Germany; 10 Institute for Neuropathology, University of Münster, Münster, Germany; 11 Department of Radiation Oncology ACTREC, Tata Memorial Centre, HBNI Kharghar, Navi Mumbai, 410210 India; 12 Department of Clinical and Molecular Medicine, Faculty of Medicine and Health Sciences, Laboratory Centre, University of Science and Technology (NTNU), St. Olavs Hospital, Norwegian, Trondheim, NO-7491 Norway; 13 Department of Pathology, Laboratory Centre, St. Olavs Hospital, Trondheim, NO-7030 Norway; 14 Department of Neuro-Oncology, City of Health and Science Hospital, University of Turin, Turin, Italy; 15 Department of Oncology, Leuven Cancer Institute, KU Leuven, Leuven, Belgium; 16 Department of Clinical Physiology, Sahlgrenska University Hospital, Gothenburg, Sweden; 17 Department of Molecular and Clinical Medicine, Institute of Medicine, Sahlgrenska Academy, Gothenburg University, Gothenburg, Sweden; 18 Instituto de Investigación Biomédica de Salamanca (IBSAL), University Hospital of Salamanca, Salamanca, Spain; 19 Department of Neurosurgery, Athens Medical Center, Athens, Greece; 20 European University of Cyprus Medical School, Cyprus, Greece; 21 Neurosurgery Service of the University Hospital of Salamanca, Salamanca, 37007 Spain; 22 Department of Neurosurgery, Yonsei University College of Medicine, Seoul, Republic of Korea; 23 Department of Radiation Oncology, Yonsei University College of Medicine, Seoul, Republic of Korea; 24 Luis Carlos Sarmiento Angulo Cancer Treatment and Research Center, Bogotá, Colombia; 25 Thoracic Oncology Unit, Instituto Nacional de Cancerología (INCaN), México City, México; 26 Department of Neurology and Chao Family Comprehensive Cancer, University of California, Irvine, Irvine, USA; 27 Allina Health, Minneapolis, MN USA; 28 Department of Clinical Medicine and Biotech Research and Innovation Center (BRIC), University of Copenhagen, Copenhagen, Denmark; 29 Department of Clinical Medicine, University of Copenhagen, Copenhagen, Denmark; 30 Deptarment of Congenital Disorders, Statens Serum Institut, Copenhagen, Denmark; 31 Department of Clinical Physiology and Nuclear Medicine, Copenhagen University Hospital-Rigshospitalet, Copenhagen, Denmark; 32 Department of Neurological Surgery, Istituto Nazionale Neurologico “C.Besta”, Milan, Italy; 33 Research Center for Medical Image Analysis and Artificial Intelligence (MIAAI), Faculty of Medicine and Dentistry, Danube Private University, Krems, 3500 Austria; 34 Division of Oncology, Department of Medicine I, Medical University of Vienna, Vienna, Austria; 35 University of Lucerne, Lucerne, Switzerland; 36 St. Anna Hospital, University of Lucerne, Lucerne, Switzerland; 37 Department of Clinical Neuroscience, Karolinska Institutet, Stockholm, Sweden

**Keywords:** Treatment-refractory, Progressive meningioma, Retrospective cohort, Individual participant data, Recommendations

## Abstract

**Background:**

*Treatment-refractory meningioma* is a widely used term but lacks standardized criteria, impairing research comparability and treatment evaluation. The aim of this study was to assess the heterogeneity of patient populations labeled as *treatment-refractory* and to explore recommendations for better consistency.

**Methods:**

We systematically reviewed 69 studies published before 2024 and analyzed individual participant data from 15 cohorts (*n* = 211) that included *treatment-refractory* patients who underwent experimental therapy with somatostatin receptor (SSTR)-targeted therapies. A reference cohort (*n* = 102) with newly diagnosed WHO-3 meningiomas was used descriptively for comparison. Progression and death were the primary endpoints. Hazard rate ratios were estimated via Poisson regression, and inter-study heterogeneity was quantified using I² statistics.

**Results:**

Definitions of treatment-refractory varied substantially across previous studies. WHO-1 patients showed high statistical inter-study variability, particularly for the long-acting SSTR-analogues group when assessing progression (I² = 81.7%) and death (I² =74.8%). Patients with *treatment-refractory* WHO-2 and WHO-3 meningioma exhibited more consistency across endpoints and SSTR-targeted therapies (I² percentages ≤ 16.0%). Risk of progression and death differed significantly by WHO grade, regardless of SSTR-targeted therapy.

**Conclusions:**

Our findings demonstrate an inconsistent use of the term *treatment-refractory* and substantial variability of effect estimates dependeing on the individual cohorts. Pooling patients across WHO grades is unfeasible for assessing treatment effects. Based on the present study and prior evidence, we outline recommendations to improve consistency in future trial design and enable more meaningful comparisons across studies. The recommendations are grouped into four categories: radiographic evaluation, endpoints, clinical core elements, and molecular classification.

**Supplementary Information:**

The online version contains supplementary material available at 10.1007/s11060-025-05154-2.

## Introduction

Some meningioma patients progress or recur despite undergoing extensive treatment, including multiple surgeries, radiosurgery and radiotherapy [1–4]. This subgroup is commonly denoted as “treatment-refractory” meningiomas because of the resilience to standard therapy. These meningioma lesions are not restricted to malignant WHO-3 patients, although frequently associated herewith, but also include aggressive WHO-1 and WHO-2 phenotypes [5–7].

Patients with such treatment-refractory meningioma may be offered experimental treatments, often based on the assumption of clinical benefit rather than evidence from prospective trials. Synthesizing evidence from these studies remains of significant interest, considering the rarity of aggressive meningiomas and the lack of effective medical treatment options. However, without a standardized inclusion framework, the external validity of the individual cohorts remains limited and interpretation of evidence from such retrospective studies is challenging due to unmeasured confounding and inherent heterogeneity. In most previous studies, parameters that affect outcomes are often not reported or considered, such as molecular characteristics or adherence to standardized criteria for radiological recurrence and progression [1, 2, 4, 8]. A better understanding of the term “treatment-refractory meningioma” is therefore essential, as it directly influences the patient populations on which outcomes of experimental treatments are assessed and compared. While the term is intuitively understandable, its extension to specific criteria that define these tumors has not been systematically addressed.

We hypothesize a significant variability in study cohorts labelled “treatment refractory meningioma”. Thus, we first aim to assess and quantify this variability in previous studies reported. Second, we aim to determine the effect of a potential variability on the external validity using cohorts of treatment-refractory meningioma patients as illustrative examples. We specifically analyzed individual participant data from 15 unique cohorts evaluating experimental somatostatin receptor (SSTR)-targeted treatments. SSTRs, particularly the SSTR2A subtype, are frequently overexpressed in meningiomas and have historically been investigated as therapeutic targets in treatment-refractory cases, making SSTR-directed therapies a commonly used experimental approach in this setting [1, 9–11]. These include data from our recent individual participant data meta-analyses on peptide receptor radionuclide therapy (PRRT) and long-acting somatostatin analogues (SSA), together representing 95% of available cohorts at the time [10, 11]. Finally, we aim to assess risks of progression and death between WHO-1, -2, and − 3 treatment-refractory meningioma vs. a reference cohort, to answer if they should be considered a single disease entity. Here, we utilized a subset of aggressive meningiomas that were recorded in our international *PERNS* database, which contains individual participant data on approximately 8,000 primary meningiomas collected world-wide. This reference group was comprised of 102 patients with primary WHO-3 meningiomas (“*Reference group*” will refer to this group). It was selected for two main reasons: their known poor prognosis and their consistency in not having received any prior meningioma treatment before inclusion.

## Materials and methods

### Definitions of treatment-refractory meningioma in published literature

We searched for relevant literature to synthesize criteria applied to define this population in previous publications. This information was extracted from relevant articles identified from searching PubMed and ClinicalTrials.gov on July 26th, 2024. The PubMed search was performed using MeSh-terms and keywords: “refractory,” “progressive,” “recurrent,” or “high-risk,”, yielding the following search string: (“refractory” OR “progressive” OR “recurrent” OR “High-risk”) AND “meningioma”).

### The PERNS database and the reference group

Since 2019, we have compiled data on meningioma patients obtained from local databases worldwide [27]. Our coverage currently comprises approximately 8,000 patients with a primary meningioma collected from 42 centers spanning six continents. The database primarily contains patient-related information, including clinical and histopathological data, which were recorded by local physicians. Within this data compilation, a total of 102 meningioma patients were diagnosed with a primary WHO-3 lesion, and were used to assess heterogeneity across study cohorts of treatment-refractory meningioma patients who received experimental SSTR-targeted treatment.

The *Reference group* was followed from the date of primary surgery. Clinical outcomes, including progression, death, or being censored alive at last follow-up, were recorded in the database. To support data availability, Supplementary Table 1 contains the following data on each patient in the *Reference Group*: age, sex, edition of WHO classification (2007 vs. 2016), extent of resection (GTR vs. STR), total radiation dose from fractionated radiotherapy in Gy, and clinical events. However, data on molecular classification is unavailable and the *Reference Group* is intended for descriptive purposes only [7, 12].

### Assessment of heterogeneity in cohorts of patients receiving experimental therapy

We included individual treatment-refractory patient data from patients receiving PRRT (86 patients from 7 cohorts [13–19]) and SSA (125 patients from 8 cohorts [20–27]).

The patient cohorts were re-analyzed to assess study heterogeneity by also utilizing the *Reference group*. First, hazard rate ratios for each study were obtained while stratified for experimental treatment (PRRT or SSA) and the individual WHO grades. We considered two outcomes: progression and death. Within each stratum, we used a Poisson regression model adjusted for age, radiotherapy as a category (yes/no; referring to adjuvant, fractionated radiotherapy), and also the individual studies as a category. Regarding age, this covariate was included as a continuous covariate and denoted age at primary surgery for the *Reference* group and age at initiation of experimental treatment for the treatment groups, presuming a linear correlation. No patients in the PRRT or SSA groups received radiotherapy concomitantly with their respective experimental treatments. Detailed covariates such as sex, Simpson grade, and molecular markers (e.g., methylation class or genomic alterations) were not consistently reported across the included cohorts and were therefore not available for analysis. While these factors are clinically relevant, the lack of harmonized reporting limited our ability to adjust for them in the regression models. There were no missing data across the remaining covariates.

The follow-up time was subsequently organized into successive 12-months intervals, i.e. a follow-up of 30 months would yield three intervals: (interval 1) 0 to 12 months, (interval 2) 12 to 24 months, (interval 3) 24 to 30 months (stop). The Poisson regression models were finally adjusted to the interval category, with the offset defined as the *log*(stop time – start time) of each interval. This adjustment ensures that the event rates are appropriately scaled by the time each subject is at risk within each interval.

The Poisson regression models were used to obtain hazard rate ratios within each WHO- and treatment-specific stratum. The purpose of estimating hazard rate ratios was to assess between-study heterogeneity, summarized using the I² statistic, rather than to compare effect sizes across studies or between cohorts. Accordingly, the hazard rate ratios are visually presented in the figures to contextualize study variability, not for comparative inference. Based on the hazard rate ratios, we quantified I²-percentages to measure study heterogeneity, which represents the extent to which the observed variability in effect sizes across studies is due to heterogeneity rather than random variation. We obtained the log hazard ratio with corresponding standard error from the Poisson regression models and subjected these parameters to a fixed-effects model, thereby obtaining the I^2^-percentages [28]. Each study was weighted in the fixed-effects model using inverse-variance. The variance is derived from the square standard error of the *log*(hazard ratio), where a smaller weight is given to studies with a larger standard error (such as fewer participants). Three categories were used to indicate low, moderate and high study heterogeneity according to common usage (< 25%, 50% and > 75%) [29].

The stastistical software *R* v 4.3 was used.

### Risks: treatment-refractory meningioma and the reference group

The absolute risk of progression was estimated using the Aalen-Johansen method. Patients were censored if alive and progression-free at the end of follow-up or at the at time of progression-free death [30]. The risk of death was estimated using the Kaplan-Meier method.

### Ethics

As the manuscript relies entirely on published and fully anonymized material, ethical approval was not required.

## Results

### Literature overview

The median cohort size of studies investigating new treatments for patients with a treatment-refractory meningioma was 20 individuals (range: 6 to 90, interquartile: 12 and 27) (Supplementary Table 2). The definition of treatment-refractory varied considerably across studies with no consistency or considerations regarding WHO grading, histopathology, prior treatment(s) and depleted treatment options, or whether treatment was initiated due to progression of an existing lesion vs. treatment initiation to prevent recurrence of removed disease. Including the studies on PRRT and SSA, we identified 33 published studies examining 17 different drugs either alone or in combination with other modalities. The distribution of WHO-grades differed across this literature, and the WHO classification used was either 2000 (*n* = 1), 2007 (*n* = 14), 2016 (*n* = 8), or not reported (*n* = 10). Three studies used direct comparisons between the group of treated patients and a control group (Supplementary Table 2). As of February 5, 2025, a total of 34 clinical trials were registered on ClinicalTrials.gov investigating experimental therapies for treatment-refractory (or “progressive”) meningioma (Supplementary Table 3).

Only three randomized prospective trials have been published. One study was terminated due to slow accrual after enrolling 15 patients over three years [31]. A single-center trial found no efficacy of mifepristone in unresectable meningiomas compared to placebo [32]. The third, a multi-center study on recurrent WHO-2 and − 3 lesions, did not show improved progression-free or overall survival with trabectedin compared to standard care. The study, however, identified that molecular classification remained an independent risk factor in patients with treatment-refractory meningioma [33].

### Description of the reference group

Patients were diagnosed according to the 2007 edition (*n* = 46) or 2016 edition (*n* = 56) of the WHO classification. The *Reference group* was followed for 529.5 person-years with a median follow-up of 42.6 months (interquartile range (IQR) Q1 and Q3: 17.8 and 89.5 months). There were 61 patients experiencing progression of the disease and 53 deaths occurred during follow-up. In total, 82 had gross total resection (Simpson Grade 1 to 3), while the remaining 20 patients had subtotal resection (Simpson Grade 4) at baseline. There were 50 males vs. 52 females with a median age of 60 years (IQR: 51 years and 70 years). Adjuvant fractionated radiotherapy was administered to 52 patients, here with a median of 60 Gy (IQR: 59 Gy and 60 Gy) (Table 1).

**Table 1:** Characteristics of the patients included in the *Reference group*

Clinical variables	Radiotherapy, *n* = 52 (%)	No radiotherapy, *n* = 50 (%)	Total, *n* = 102 (%)
Age, years (median, IQR)	58 (52–67)	63.5 (49–71)	60 (51–70)
Female sex	24 (46%)	28 (56%)	52 (51%)
WHO classification 2007 ed.	23 (44%)	23 (46%)	46 (45%)
WHO classification 2016 ed.	29 (56%)	27 (54%)	56 (55%)
STR	13 (25%)	7 (14%)	20 (20%)
GTR	39 (75%)	43 (86%)	82 (80%)
Total Gy (median, IQR)	60 (59–60)	0	-

GTR; gross total resection, STR; subtotal resection, IQR; interquartile range denote the 1st and 3rd quartile

### Description of the PPRT and SSA cohorts

**Fig. 1:**
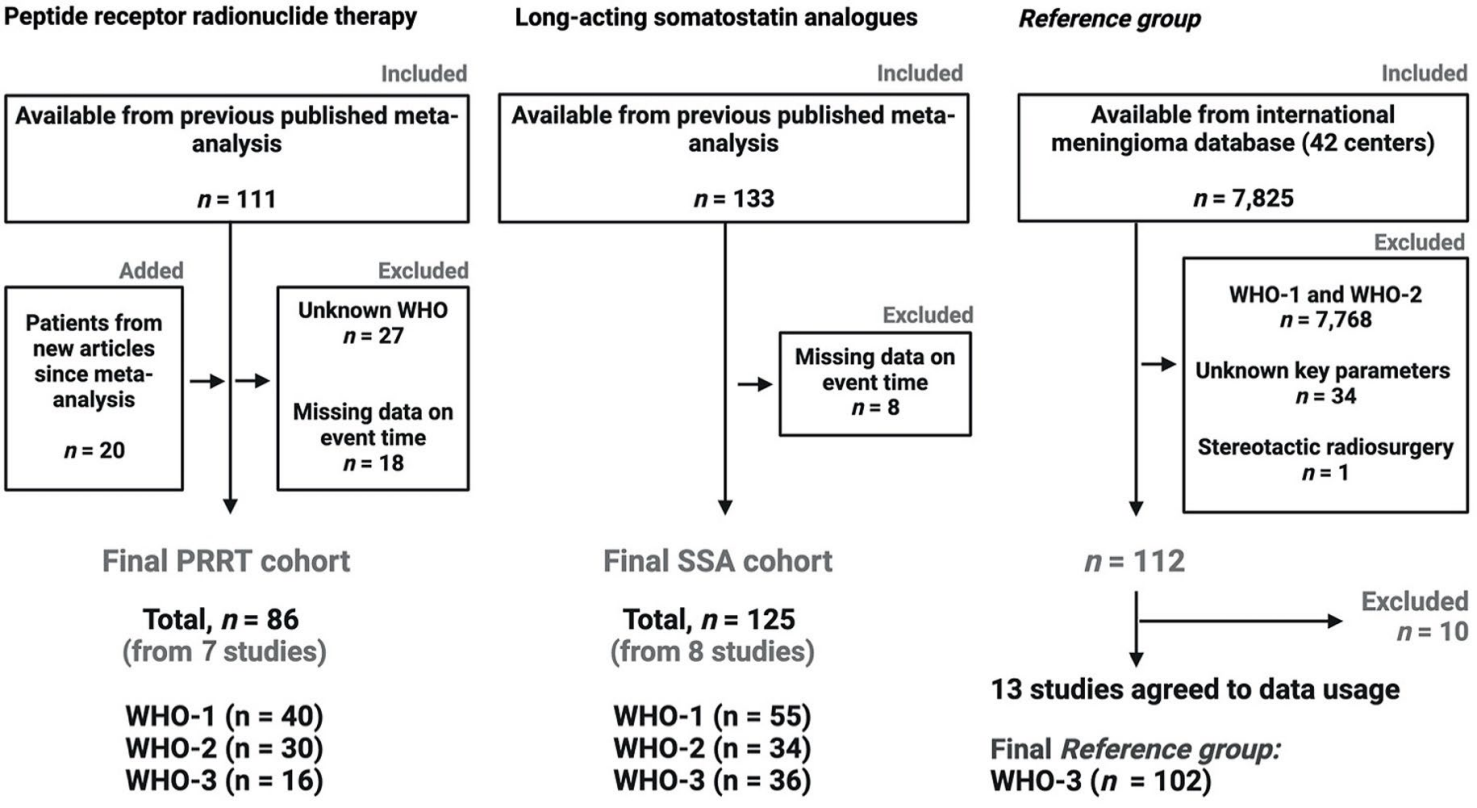
Flow chart of the study cohort, which was divided into two treatment groups (Group **A** and Group **B**) and the *Reference group.*

A flowchart outlining the three cohorts is presented in Fig. 1. There were no patients lost to follow-up in either treatment group. In total, 86 patients received PRRT and were followed for 209.0 person-years with a median follow-up of 17.2 months (IQR: 9.8 and 32.0 months). There were 40, 30, and 16 patients with WHO-1, WHO-2, and WHO-3 lesions, with 49 patients experiencing disease progression and 38 patients dying during follow-up. A total of 125 patients received SSA. The group was followed for 246.0 person-years, with a median follow-up of 19.0 months (IQR: 7.0 and 28.7 months). There were 55, 34, and 36 patients with WHO-1, WHO-2, and WHO-3 lesions, with 79 experiencing disease progression and 46 dying during follow-up.

### Study heterogeneity by WHO grade and treatment type

**Fig. 2:**
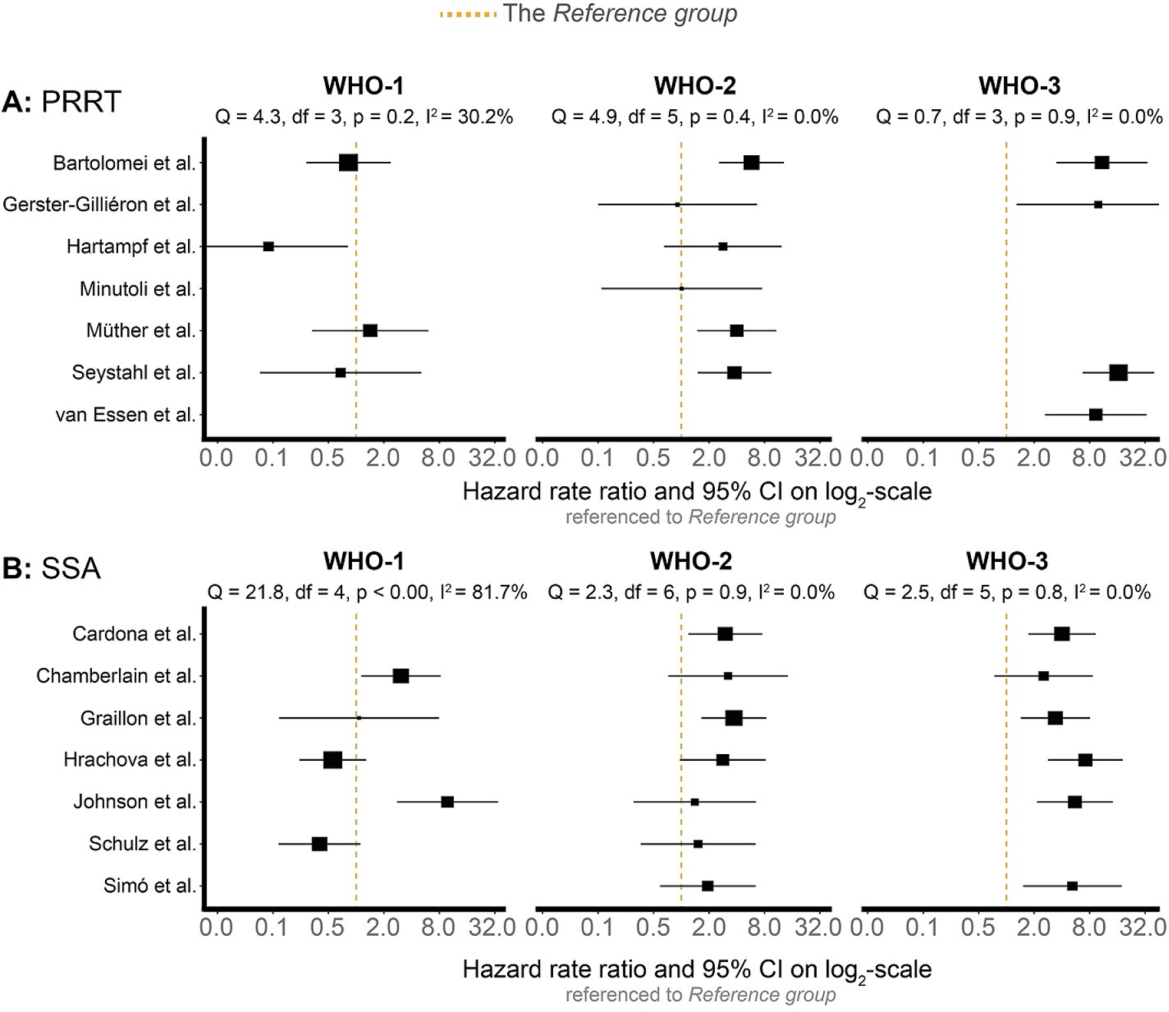
The risk of progression presented as hazard rate ratios for each of the included studies when compared with the *Reference group*. Patients stratified for type of treatment and WHO-grade. A fixed-effects model on *log*(hazard ratios) and corresponding standard errors were undertaken to render I^2^-percentages. Each square reflects the weight given to the individual study (inverse-variance). Variance is derived from the square standard error of the *log*(hazard ratio). Inverse-variance reflect 1/variance, i.e., smaller weight is given to studies with a larger standard error, typically directly related to fewer participants. *Cardona et al. and Graillon et al. combined SSA with Everolimus [20, 23]. **A**: Peptide receptor radionuclide therapy. **B**: Long-acting somatostatin analogues

**Fig. 3:**
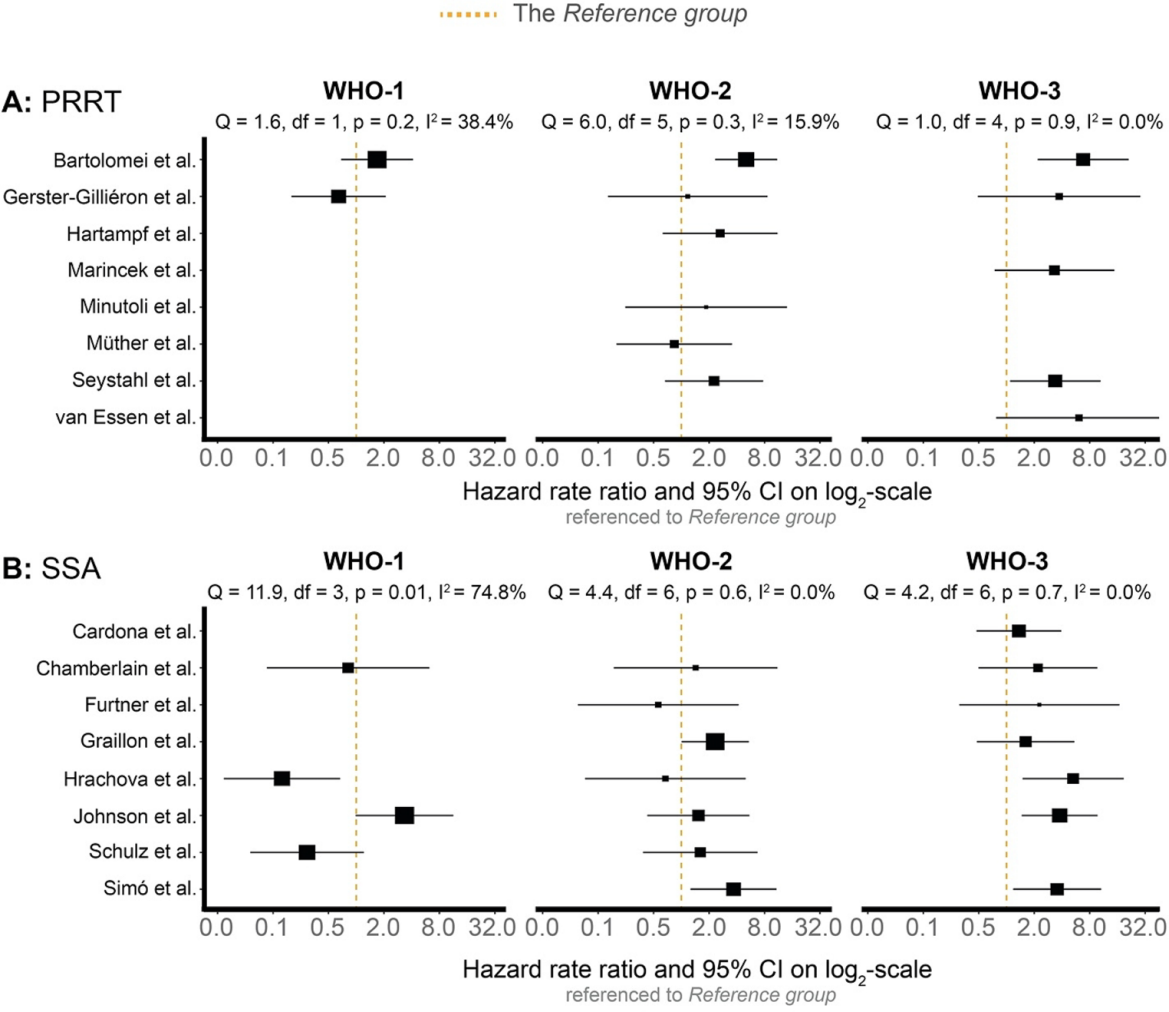
The risk of death presented as hazard rate ratios for each of the included studies when compared with the *Reference group*. Patients stratified for type of treatment and WHO-grade. A fixed-effects model on *log*(hazard ratios) and corresponding standard errors were undertaken to render I^2^-percentages. Each square reflects the weight given to the individual study (inverse-variance). Variance is derived from the square standard error of the *log*(hazard ratio). Inverse-variance reflect 1/variance, i.e., smaller weight is given to studies with a larger standard error, typically directly related to fewer participants. *Cardona et al. and Graillon et al. combined SSA with Everolimus [20, 23]

The I²-percentages obtained suggested that WHO-2 and − 3 treatment-refractory meningioma were associated with (very) low and insignificant heterogeneity in both the PRRT and SSA treatment groups, regardless of outcome (Figs. 2 and 3). Specifically, when progression was the outcome, heterogeneity was 0% for both WHO-2 and WHO-3 in both treatment groups. The same was true for death, except for WHO-2 patients receiving PRRT, where a modest I² of 15.9% was observed. Contrarily, treatment-refractory WHO-1 meningioma displayed moderate to high heterogeneity. Specifically, WHO-1 patients receiving PRRT showed no significant heterogeneity; however, the heterogeneity reported was moderate with I^2^-percentages of 30.2% (*P* = 0.2) and 38.4% (*P* = 0.2) for progression and death as outcomes (Figs. 2A and 3A). For the SSA group, WHO-1 patients indicated significant inconsistencies in both progression (I^2^ = 81.7%, *P* < 0.01, Fig. 2B) and death (I^2^ = 74.8%, *P* = 0.01, Fig. 3B).

### Risks of progression and death

**Fig. 4:**
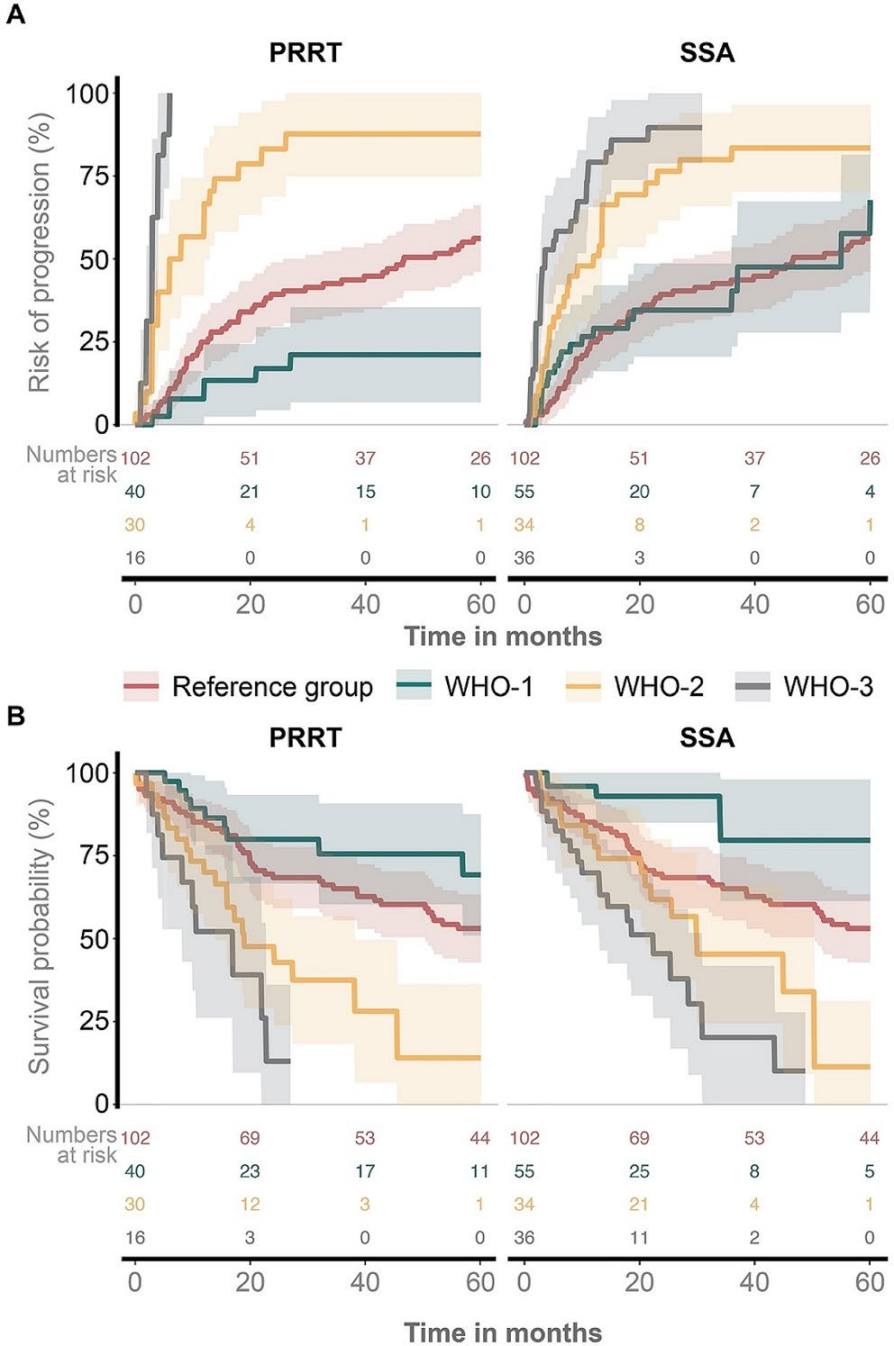
**A**: Aalen-Johansen estimates - risk of progression. **B**: Kaplan-Meier estimates – overall survival probability. *PRRT: peptide receptor radionuclide therapy; *SSA: long-acting somatostatin analogues

For WHO-2 and WHO-3 patients, the absolute risk of progression was substantially higher than for the *Reference group*, regardless of whether PRRT or SSA was administered (Fig. 4AB). Conversely, compared to the *Reference group*, WHO-1 patients with treatment-refractory meningioma had a lower absolute risk of progression in the PRRT cohort. At the same time, those receiving SSA displayed similar outcomes to the *Reference group*. As shown in Fig. 4CD, the risk of death followed a pattern similar to the absolute risk of progression.

## Discussion

Studies assessing the effects of experimental therapies often include patients with treatment-refractory meningiomas, both in previous literature and in ongoing trials. We identified 69 of such studies published or ongoing between 2010 and early 2025. These studies typically offered salvage or compassionate-use treatments after conventional therapies failed.

### Treatment-refractory meningioma is not one disease entity

Studies on treatment-refractory meningiomas often pool patients regardless of WHO grade (Supplementary Tables 2 and 3), thus assuming similar prognostic impact across grades. A key finding was that discrimination between the individual WHO grades remained after pooling of data, suggesting that treatment-refractory meningiomas should *not* be considered as a single disease category but distinguished. Consequently, if data are pooled, the estimates reported from such studies will partially reflect the underlying distribution of WHO grades.

### Definition of “treatment-refractory” disease

Across the 69 studies, inclusion and exclusion criteria varied widely, with no consistent or traceable definition of treatment-refractory meningioma. Investigating novel therapies is futile if external validity is undermined by fundamentally different patient populations. The potential benefits of new treatment strategies could be missed if studies that appear similar in design yield contradictory findings because the fundamental differences in baseline characteristics of their populations are unrecognized, rather than true inconsistent treatment effects. Variability of what constitutes “treatment-refractory” allows inclusion of heterogenous subgroups where heterogeneity can obscure essential effects that would be visible in sharper defined subgroups.

### Study heterogeneity

By utilizing the *Reference group*, we used studies that previously applied SSTR-targeted therapies to assess study heterogeneity. There was statistically insignificant and low-to-moderate heterogeneity among WHO-2 and − 3 treatment-refractory meningiomas, i.e. the progression rate and mortality rate were similar across the individual studies including the WHO-2 and WHO-3 grades. The WHO-1 subgroup, in contrast, showed moderate-to-high and statistically significant I²-percentages across the studies. This could indicate that WHO-1 treatment-refractory patients are more inconsistently defined across studies than their WHO-2 or WHO-3 counterparts. While WHO grade appears to be a dominant contributor to outcome variability, additional factors such as differences in imaging intervals, timing of salvage therapy initiation, and local treatment algorithms may also play important roles. These unmeasured variables likely compound the observed heterogeneity and reinforce the need for more detailed and standardized data collection in future trials.

### Recommendations

We integrate findings from this present study with recommendations from previous trials and guidelines to enhance the consistency and clarity in defining “treatment-refractory” meningiomas. Four main areas were considered and summarized in Table 2: (1) definition, (2) endpoints, (3) clinical core elements and, (4) molecular classification.

**Table 2:** Recommendations for “treatment-refractory meningioma”

Framework	Recommendation
Definition	Radiographic criteria, eligible for clinical trial enrollment as per RANO and EORTC-1320 trial [4,33]: • “A 15% increase in the sum of the products of perpendicular diameters of the contrast-enhancing lesions within the prior 6 months” • Measurable disease (minimum 10 mm × 10 mm) on MRI at baseline without extracranial metastasis. Baseline assessments should be performed as close to treatment initiation as possibly, preferably within two weeks prior to treatment initiation/randomization, and not more than 4 weeks. Clinical criteria • Depleted treatment options in terms of surgery and radiotherapy. This criteria is highly site-specific and depend on numerous factors that cannot be generalized, emphasizing the importance of reporting these parameters in “Core elements” • No prior systemic or experimental therapy has been applied.
Endpoints	Radigraphic response criteria are defined in the RANO group, and include: complete response, partial response, minimal response, stable disease and, progressive disease [4]. We recommend evaluating treatment response at 6- and 12-months post-treatment, in alignment with previous endpoints. Historical benchmarks of endpoints should be used with caution, and only for descriptive purposes – not analytical nor to derive conclusions.
Core elements	Experimental therapy-level module: proposed data elements to report • Agent used & mechanism of action • Toxicities • Definition of response criteira o Preferably adheres to RANO response criteria [4] • Criteria for termination of experimental therapy • Cumulative dose • Documentation of “depleted treatment options” o Number of surgeries o Radiotherapy (total dose in Gy, fractionation) o Radiosurgery (total dose in Gy, target area in cm³)
Molecular classification	Implementation of molecular classification, such as DNA methylation, will provide better objective metrics for adjustment and comparison of baseline prognosis. If molecular classification is not feasible, differentiation between individual WHO grades is recommended due to persistent differences in risk profiles.

#### Definition

A critical element is to more accurately assess radiologically confirmed tumor progression before starting treatment, minimizing bias from different growth kinetics. The Response Assessment in Neuro-Oncology (RANO) Working Group proposed a definition of progression to allow for clinical trial enrollment: “… patients be considered eligible for clinical trials if there is 15% increase in the sum of the products of perpendicular diameters of the contrast-enhancing lesions within the prior 6 months” [4]. As discussed in the EORTC-1320 trial, a treatment-refractory meningioma should specifically denote visible residuals – not gross totally removed lesions – measurable (minimum 10 mm × 10 mm) on MRI within four weeks prior to treatment initiation/randomization [4, 33]. Moreover, cases with extracranial metastasis should not be included.

Standard criteria should include patients who have exhausted conventional treatment options, specifically surgery and radiotherapy. However, this is difficult to generalize, as such considerations are site-specific and influenced by multiple factors, including local guidelines, clinical practices, and individual physician decisions. However, these criteria are essential for defining clinical eligibility as “treatment-refractory”. Given the vast potential for inconsistencies, these critical parameters should be clearly detailed in a “Clinical core elements” section (discussed below). Finally, eligible patients must not have previously received systemic therapy for their meningioma [4, 33, 34].

#### Endpoints

Kotecha et al. recently benchmarked the efficacy of salvage therapy for recurrent meningiomas concerning PFS-6 (progression-free survival at 6 months) and PFS-12 [2]. These benchmarks were derived from a systematic review and meta-analysis of available clinical trials, incorporating pooled PFS rates to establish efficacy thresholds for future trials.

While benchmarks can provide useful reference points for evaluating salvage therapies in recurrent meningiomas, their applicability is highly context-specific and depends on the patient populations. The absence of a universal definition for treatment-refractory meningioma and the frequent, yet misleading, random pooling of WHO grades (e.g., WHO-2/-3) perpetuate significant variability and study heterogeneity. A key limitation is selection bias and related unmeasured confounders in the studies forming the basis of these benchmarks, ultimately reducing their external validity. If study populations are not representative of the broader treatment-refractory meningioma population, these benchmarks may not translate well to future trials. Arguably, no such “representative cohort” of treatment-refractory meningioma exists, and the benchmark estimates are, as such, derived from non-representative cohorts that may ultimately obscure actual treatment effects.

We recommend adapting the proposed assessment scheme by the RANO Working Group that encompass: complete response, partial response, minimal response, stable disease, and progressive disease. Specifically, radiographic progression is defined as an “increase by ≥ 25% in sum of the products of perpendicular diameters of target lesions compared with the smallest tumor measurement obtained either at baseline (if no decrease) or best response”; or, if new lesions develops, that are measurable in at least two projections [4, 34]. To ensure consistency in evaluating tumor progression and comparability across studies, radiographic assessments should be obtained at standardized time points for all patients – e.g., in 6-months intervals, but not less [4].

#### Clinical core elements

Nassiri et al. recently provided a framework for clinical core data elements in studies on meningiomas to improve comparability but did not address experimental therapy or treatment-refractory meningioma [35]. The current framework considers two modules: the patient-level module (including e.g. age, sex, receipt of prior chemotherapy, prior cranial radiation and chemotherapy, history of malignancy and multiple meningiomas, date of death) and the tumor-level module, which include timing of surgery, tumor size and location, resection extent/Simpson grade, histopathological grade [36], year of WHO classification, and time to recurrence [35]. We propose an additional third module, specifically an experimental therapy-level module. The experimental therapy-level module should contain data elements on: agent used and mechanism of action, toxicities, definition of progression (preferably adherence to RANO response criteria [4]), termination of experimental therapy in case of clinical deterioration, and cumulative dose at either recurrence/progression, death or study termination. Finally, transparency of what defined “depleted treatment options” locally is necessary to specify the treatments to which individual patients have become refractory, including the number of surgeries, radiotherapy (total dose in Gy and fractionation), and radiosurgery (total dose in Gy and target area in cm^3^).

#### Molecular classification

While not yet routinely implemented, molecular classification methods such as DNA methylation profiling may offer a more objective and reproducible framework for stratifying meningiomas, particularly in aggressive subtype [7, 8, 12, 33, 36, 37]. Ideally, these markers would constitute an independent parameter within the experimental therapy-level module of the “core elements. In the future, broader adoption of such techniques could enhance consistency in patient selection, risk stratification, and trial comparability. However, most current and ongoing trials have yet to systematically incorporate or report molecular data. Until molecular classification is implemented widely, harmonization efforts must rely on accurate histopathological grading. As demonstrated in this study, distinguishing between individual WHO grades remains essential for ensuring clinical and methodological clarity in the absence of molecular criteria.

### Strengths and limitations

The presented data compilation is exceptionally comprehensive and allowed for a detailed analysis of heterogeneity at the study level by utilizing unique individual participant data from 211 treatment-refractory meningioma patients. No formal power or sample-size calculation was performed, as the study is retrospective, descriptive, and exploratory in nature, without a predefined effect-size hypothesis. The pooled sample reflects available international patient-level data from previously published cohorts and was not assembled to support inferential comparisons. Rather, the aim was to illustrate the conceptual and outcome-related heterogeneity in how the label *treatment-refractory meningioma* has been operationalized across studies.

A limitation in navigating information collected from aggressive meningiomas is the frequent change of disease definitions and increased knowledge of molecular mechanisms with associated classification. As such, the lack of molecular data is a limitation; however, data from the patients we studied were largely obtained before molecular information and methylation profiling were introduced to risk stratify patients [38–41]. Incorporating molecular information might have allowed for a more detailed and comprehensive assessment of patients with such lesions. In addition, key covariates such as sex and extent of resection (like Simpson grade) were not consistently available across cohorts, and therefore not included in our adjustment of confounders. We cannot exclude the possibility that adjustment for these variables may have influenced the estimated hazard rate ratios, highlighting the importance of complete and standardized data collection in future research.

Although RANO criteria represent a standardized framework for defining progression in meningioma, they were not used as inclusion criteria across the included studies [4]. As such, we were unable to uniformly apply or report a single definition of progression, further illustrating the heterogeneity in the source literature and the challenges it poses to meaningful comparison.

Finally, the WHO grading is another source of imprecision. The WHO 2021 classification introduced molecular features and histopathological refinements that may have led to reclassification of some lesions previously classified as WHO-1 or WHO-2. Although our dataset primarily includes patients categorized under the 2007 and 2016 editions, literature suggests that between 3.9% and 26.5% of cases may be reclassified using the 2021 criteria [38–41]. This highlights the need for harmonization across WHO editions and consistent classification approaches in future treatment-refractory meningioma research.

## Conclusions

The term “treatment refractory meningioma” is frequently used in published and ongoing studies. While the term itself is intuitively clear, our analysis revealed significant variability in its usage, consequently affecting external validity. The risks of progression and death differed between WHO-1, -2, and − 3 lesions, suggesting that treatment-refractory meningioma should not be considered a single disease entity. Therefore, we do not recommend pooling of data across WHO grades in future studies. To improve clarity and consistency in the use of the term “treatment-refractory,” we propose recommendations summarized from previous trials and guidelines while also incorporating findings from this study.

## Electronic supplementary material

Below is the link to the electronic supplementary material.

Supplementary Material 1

Supplementary Material 2

Supplementary Material 3

## Data Availability

No datasets were generated or analysed during the current study.
