# Supplementary material for: The concept, intention, and evaluation of the term treatment-refractory meningioma
Source: J Neurooncol. 2025 Aug 4;175(2):599–610. doi: 10.1007/s11060-025-05154-2 (PMC12420741; doi:10.1007/s11060-025-05154-2)
Supplement: Supplementary file 1 — Supplementary Material 1 [file 11060_2025_5154_MOESM1_ESM.docx]

**Supplementary Table 2**. Summary of studies on treatment-refractory meningioma cohorts from 2010 and onwards.

| Published since 2010 | | | | | | |
| --- | --- | --- | --- | --- | --- | --- |
| **Study** | **Drug** | **Mechanism of action** | **Cohort** | **WHO classification** | **Definition of ’treatment refractory’/inclusion criteria** | **Reference group** |
| Chamberlain 2011[1] | Hydroxyurea | Ribonucleotide reductase inhibitor | WHO-1 (n = 60) | Not reported | Surgery and radiation refractory WHO grade 1 recurrent meningiomas | No |
| Chamberlain 2012[2] | Hydroxyurea | Ribonucleotide reductase inhibitor | WHO-2 (n = 22)  WHO-3 (n = 13) | Not reported | Patients all treated by the author with WHO Grade 2 or 3 recurrent meningiomas following prior surgery and radiotherapy | No |
| Mazza 2016[3] | Hydroxyurea or Hydroxyurea + Imatinib | Ribonucleotide reductase inhibitor and VEGFR | WHO-1 (n = 2)  WHO-2 (n = 9)  WHO-3 (n = 1)  N/A  (n = 3) | 2007 | Recurrent or progressive meningioma of any grade, not amenable to surgery, radiotherapy or radiosurgery, were eligible for this trial | Comparing Hydroxyurea vs hydroxyurea + imatinib |
| Karsy 2016[4] | Hydroxyurea + verapamil | Ribonucleotide reductase inhibitor + Calcium antagonist | WHO-1 (n = 2)  WHO-2 (n = 5) | 2000 | Histologically confirmed meningioma of any WHO grade that demonstrated radiographic progression of at least 25% increase in tumor cross-sectional area measured on computed tomography or MRI studies within the last 6 months and recurrence after surgery or radiotherapy. | No |
| Johnson 2011[5] | Octreotide | Long-acting somatostatin analogue | WHO-1 (n = 3)  WHO-2 (n = 3)  WHO-3 (n = 5) | 2007 | Recurrent or progressive biopsy-proven unresectable meningioma. the tumor must have progressed despite prior radiotherapy or the patient was not a candidate for radiotherapy or gamma knife radiotherapy | No |
| Norden 2015[6] | Octreotide | Long-acting somatostatin analogue | WHO-1 (n = 9)  WHO-2 (n = 12)  WHO-3 (n = 6) | Not reported | Histologically confirmed recurrent or progressive meningioma of any grade. No limit on the number of previous therapies | No |
| Cardona 2019[7] | Octreotide + everolimus | Long-acting somatostatin analogue + mTOR inhibitor | WHO-2 (n = 6)  WHO-3 (n = 8) | 2016 | Histopathologically confirmed grade II or III meningioma. Progressive or recurrent tumor after prior therapy (surgical resection and/or radiation therapy). | No |
| Furtner 2016[8] | Octreotide (1 case: + lanreotide) | Long-acting somatostatin analogue | WHO-2 (n = 7)  WHO-3 (n = 1) | 2007 | Histological diagnosis of WHO grade II or grade III meningioma, and received systemic antineoplastic therapy for tumor recurrence after previous operation and/or radiotherapy | No |
| Graillon 2020[9] | Octreotide + everolimus | Long-acting somatostatin analogue + mTOR inhibitor | WHO-1 (n = 2)  WHO-2 (n = 10)  WHO-3 (n = 8) | 2016 | Histologically confirmed meningioma of grade I, II, or III, and ineligible for further surgery/ radiotherapy. In addition, inclusion required a documented progression based on two different MRIs performed before inclusion | No |
| Hrachova 2020[10] | Octreotide | Long-acting somatostatin analogue | WHO-1 (n = 31)  WHO-2 (n = 5)  WHO-3 (n = 6) | 2016 | Recurrent and/or progressive meningioma expressing sandostatin receptors. Patients were determined to be poor candidates for surgical resection, stereotactic radiosurgery, or radiation therapy based on tumor location, increased risk factors for postoperative morbidity and mortality, or individual preference for non-invasive approach, or were shown to have recurrence despite surgical or radiation therapy. Patients who suffered from meningioma recurrence were offered treatment independent of history of prior surgeries, chemotherapy, radiation, or radiosurgery treatments | No |
| Schulz 2011[11] | Octreotide | Long-acting somatostatin analogue | WHO-1 (n = 11)  WHO-2 (n = 2) | 2007 | Progressive residual meningiomas (according to MR imaging criteria) following operative therapy | No |
| Simó 2014[12] | Octreotide | Long-acting somatostatin analogue | WHO-2 (n = 5)  WHO-3 (n = 4) | 2007 | Chemotherapy-naïve adult patients with a histologically confirmed diagnosis of high-grade meningioma (WHO grade II or III) and progression after prior surgery and radiotherapy. Non-eligibility for safety re-resection or re-irradiation | No |
| Minutoli 2014[13] | ^111^In-Pentetreotide labeled | Somatostatin receptor–targeted radiopeptide | WHO-1 (n = 5)  WHO-2 (n = 3) | Not reported | Inoperable somatostatin receptor positive meningiomas referred to the section of Nuclear Medicine of our University Hospital with a diagnosis of meningioma | No |
| Marincek 2015[14] | ^90^Y-DOTATOC or ^177^Lu-DOTATOC | Somatostatin receptor–targeted radiopeptide | WHO-2 (n = 6)  WHO-3 (n = 3)  N/A  (n = 20) | 2007 | Progressive unresectable meningioma. Histologically confirmed meningioma, disease progression within 12 months before study entry | No |
| Gerster-Gilliéron 2015[15] | ^90^Y-DOTATOC | Somatostatin receptor–targeted radiopeptide | WHO-1 (n = 9)  WHO-2 (n = 2)  WHO-3 (n = 1)  N/A  (n = 3) | 2007 | Recurrent or progressive meningiomas in functionally critical areas, or unfavorable medical risk profile or refusal of surgery | No |
| Seystahl 2016[16] | ^177^Lu-DOTATATE or ^90^Y-DOTATOC | Somatostatin receptor–targeted radiopeptide | WHO-1 (n = 5)  WHO-2 (n = 7)  WHO-3 (n = 8) | 2007 | Meningiomas that progressed after several lines of therapy | No |
| Hartrampf [17]2020 | ^177^Lu-DOTATATE/-TOC and EBRT | Somatostatin receptor–targeted radiopeptide | WHO-2 (n = 6)  WHO-3 (n = 2)  N/A (n = 2) | 2007 | Unresectable advanced primary or recurrent meningioma | No |
| Müther 2020[18] | ^177^Lu-DOTATATE | Somatostatin receptor–targeted radiopeptide | WHO-1 (n = 2)  WHO-2 (n = 5) | 2016 | Progressive intracranial meningioma |  |
| Lou 2012[19] | Bevacizumab | Anti-VEGF antibody | WHO-1 (n = 5)  WHO-2 (n = 5)  WHO-3 (n = 3)  N/A (n = 1) | 2007 | Recurrent/progressive meningioma following resection and radiotherapy | No |
| Nayak 2012[20] | Bevacizumab | Anti-VEGF antibody | WHO-2 (n = 6)  WHO-3 (n = 9) | 2007 | Failed maximal surgical resection and radiation and histological diagnosis of WHO Grade II or III meningioma | No |
| Furuse 2015[21] | Bevacizumab | Anti-VEGF antibody | WHO-1 (n = 2)  WHO-2 (n = 1)  WHO-3 (n = 3) | Not reported | All patients had undergone surgical resection and multiple sessions of radiotherapy, and recently developed massive perilesional edema. There were three non-irradiated tumors that had developed after radiotherapy in two patients with anaplastic meningiomas. These tumors were defined as “non-irradiated tumors.” All patients had tumors that had been previously treated with radiotherapy. | No |
| Shih 2015[22] | Bevacizumab | Anti-VEGF antibody | WHO-1 (n = 4)  WHO-2 (n = 7)  WHO-3 (n = 5)  N/A (n = 1) | 2007 | Symptomatic WHO grades I, II, or III progressive or refractory meningioma for which they had received up to one prior systemic regimen were enrolled. Patients must have undergone surgical resection, if possible, and definitive radiotherapy, when appropriate, for unresectable or recurrent disease. | No |
| Kumthekar2022 [23] | Bevacizumab | Anti-VEGF antibody | WHO-1 (n = 10)  WHO-2 (n = 10)  WHO-3 (n = 12) | 2016 | Surgery and radiation-refractory meningioma. Prior histologically proven meningioma and have unequivocal radiographic evidence of tumor recurrence or progression. | No |
| Alexander 2022[24] | Bevacizumab | Anti-VEGF antibody | WHO-1 (n = 2)  WHO-2 (n = 10)  WHO-3 (n = 11) | Not reported | Recurrent cranial meningioma following surgery, chemotherapy and radiation | No |
| Raizer 2010[25] | Vatalanib | VEGFR + PDGFR TKI | WHO-2 (n = 14)  WHO-3 (n = 7) | Not reported | Meningioma with unequivocal evidence of tumor progression or recurrence on cranial imaging. Patients having undergone resection of their tumor at recurrence or progression were eligible after they recovered from surgery | No |
| Kaley 2015[26] | Sunitinib | VEGFR + PDGFR TKI | WHO-2 (n = 30)  WHO-3 (n = 6) | Not reported | Patients were required to have histologically proven meningioma or classic radiographic features of surgically inaccessibility. All patients had to have recurred despite radiotherapy, unless radiotherapy was contraindicated. There was no limit on the number of prior surgeries, radiation or radiosurgery treatments, or chemotherapy regimens. | No |
| Reardon 2012[27] | Imatinib + Hydroxyurea | VEGFR + Ribonucleotide reductase inhibitor | WHO-1 (n = 8)  WHO-2 (n = 9)  WHO-3 (n = 4) | 2007 | Histologically confirmed meningioma that was radiographically progressive or recurrent after prior surgical resection. | No |
| Ji 2015[28] | Mifepristone | Antiprogestin | WHO-2 (n = 8)  N/A (n = 72) | Not reported | Histologically confirmed primary, recurrent, or residual unresectable meningioma were eligible if they had measurable or evaluable disease by CT or MR imaging, received radiotherapy for the disease at least 1 year before study enrollment (unless radiotherapy was either inappropriate because of tumor location or declined by patient), documented evidence of disease recurrence or progression within 2 years of random assignment. | Placebo group |
| Belanger 2022[29] | Temozolomide + radiotherapy | Alkylating and radiosensitizing cytostatic | WHO-1 (n = 2)  WHO-2 (n = 8)  WHO-3 (n = 2) | 2016 | High‑grade and recurrent meningioma. All patients had at least 1 prior surgical resection of their primary tumor | Historical controls from 32 papers (n = 2585). No head-to-head comparison, but chi^2^ test applied to published estimates. |
| Brastianos 2022[30] | Pembrolizumab | PD-1 inhibitor | WHO-2 (n = 23)  WHO-3 (n = 3) | 2016 | Eligible patients had histologically confirmed progressive or residual intracranial or metastatic grade-2 or -3 meningioma. Patients must have had progressive or residual measurable disease immediately prior to enrollment. Progressive disease was defined as an increase in size of a measurable meningioma on MRI by greater than 25% (bidirectional area) on scans separated by no more than 24 months. Residual measurable disease was defined by the presence of measurable disease, or a meningioma with clearly defined margins and a minimum diameter of 10mm in one dimension, following surgery. Multifocal disease was allowed if one lesion met the criteria for measurable disease and progressive disease. Metastatic meningiomas, as defined by the presence of extracranial meningiomas, were allowed. | No |
| Yust-Katz[31] | Pembrolizumab | PD-1 inhibitor | N/A (n = 10) | Not reported | Refractory atypical/anaplastic meningioma. | No |
| Bi[32] | Nivolumab | PD-1 inhibitor | WHO-2 (n = 18)  WHO-3 (n = 7) | 2016 | Histologically confirmed grade 2 or 3 meningioma that had progressed after maximum safe resection and prior radiation therapy. There was no limit on the number of prior progressions or treatments although patients were required to have evidence of progression at least 12 weeks after prior radiation therapy and 4 weeks or 5 half-lives (whichever was shorter) from prior systemic therapy | No |
| Preusser[33] | Trabectedin | Tetrahydroisoquinoline alkaloid. Binds to DNA and affects several transcription and repair factors. | WHO-2 (n = 29)  WHO-3 (n = 61) | 2007 | Local histological diagnosis of WHO grade 2 (atypical, chordoid, clear cell) or grade 3 (papillary, rhabdoid, anaplastic/malignant) meningioma radiologically documented progression of any existing tumor (estimated planar growth >25% in the last year) or appearance of new lesions (including intra- and extra-cranial sites). Other eligibility criteria included patients with no more options for local therapy (resection or radiotherapy). | Control (standard local of care) |
| Bi [34] | Nivolumab | PD-1 inhibitor | ,Progressive WHO-2 or  WHO-3  (n = 25) |  | Histologically confirmed grade 2 or 3 meningioma that had progressed after maximum safe resection and prior radiation therapy and had a Karnofsky score of at least 70 as well as adequate organ function. There was no limit on the number of prior progressions or treatments although patients were required to have evidence of progression at least 12 weeks after prior radiation therapy and 4 weeks or 5 half-lives (whichever was shorter) from prior systemic therapy | No |
| Brastianos [35] | Pembrolizumab | PD-1 inhibitor | WHO-2 (n = 23)  WHO-3 (n = 3) |  | Eligible patients had histologically confirmed progressive or residual intracranial or metastatic grade-2 or -3 meningioma. Based on inclusion criteria for prior trials for treatment-refractory meningiomas and consensus RANO recommendations, grade-2 and -3 meningiomas were included. Patients must have had progressive or residual measurable disease immediately prior to enrollment. Progressive disease was defined as an increase in size of a measurable meningioma on MRI by greater than 25% (bidirectional area) on scans separated by no more than 24 months. Residual measurable disease was defined by the presence of measurable disease, or a meningioma with clearly defined margins and a minimum diameter of 10 mm in one dimension, following surgery. Multifocal disease was allowed if one lesion met the criteria for measurable disease and progressive disease. Metastatic meningiomas, as defined by the presence of extracranial meningiomas, were allowed. |  |

**REFERENCES**

[1] Chamberlain MC, Johnston SK. Hydroxyurea for recurrent surgery and radiation refractory meningioma: a retrospective case series. J Neurooncol 2011;104:765–71. https://doi.org/10.1007/s11060-011-0541-5.

[2] Chamberlain MC. Hydroxyurea for recurrent surgery and radiation refractory high-grade meningioma. J Neurooncol 2012;107:315–21. https://doi.org/10.1007/s11060-011-0741-z.

[3] Mazza E, Brandes A, Zanon S, Eoli M, Lombardi G, Faedi M, et al. Hydroxyurea with or without imatinib in the treatment of recurrent or progressive  meningiomas: a randomized phase II trial by Gruppo Italiano Cooperativo di Neuro-Oncologia (GICNO). Cancer Chemother Pharmacol 2016;77:115–20. https://doi.org/10.1007/s00280-015-2927-0.

[4] Karsy M, Hoang N, Barth T, Burt L, Dunson W, Gillespie DL, et al. Combined Hydroxyurea and Verapamil in the Clinical Treatment of Refractory  Meningioma: Human and Orthotopic Xenograft Studies. World Neurosurg 2016;86:210–9. https://doi.org/10.1016/j.wneu.2015.09.060.

[5] Johnson DR, Kimmel DW, Burch PA, Cascino TL, Giannini C, Wu W, et al. Phase II study of subcutaneous octreotide 2011;13:530–5.

[6] Norden AD, Ligon KL, Hammond SN, Muzikansky A, Reardon DA, Kaley TJ, et al. Phase II study of monthly pasireotide LAR (SOM230C) for recurrent or progressive meningioma. Neurology 2015;84:280–6. https://doi.org/10.1212/WNL.0000000000001153.

[7] Cardona AF, Ruiz-Patiño A, Zatarain-Barrón ZL, Hakim F, Jiménez E, Mejía JA, et al. Systemic management of malignant meningiomas: A comparative survival and molecular marker analysis between Octreotide in combination with Everolimus and Sunitinib. PLoS One 2019;14:1–13. https://doi.org/10.1371/journal.pone.0217340.

[8] Furtner J, Schöpf V, Seystahl K, Le Rhun E, Rudà R, Roelcke U, et al. Kinetics of tumor size and peritumoral brain edema before, during, and after systemic therapy in recurrent WHO grade II or III meningioma. Neuro Oncol 2016;18:401–7. https://doi.org/10.1093/neuonc/nov183.

[9] Graillon T, Sanson M, Campello C, Idbaih A, Peyre M, Peyriere H, et al. Everolimus and octreotide for patients with recurrent meningioma: Results from the phase II CEVOREM trial. Clinical Cancer Research 2020;26:552–7. https://doi.org/10.1158/1078-0432.CCR-19-2109.

[10] Hrachova M, Nguyen ENT, Fu BD, Dandekar MJ, Kong XT, Cadena G, et al. A Retrospective Interventional Cohort Study to Assess the Safety and Efficacy of Sandostatin LAR for Treatment of Recurrent and/or Refractory Meningiomas. Front Neurol 2020;11:1–11. https://doi.org/10.3389/fneur.2020.00373.

[11] Schulz C, Ulm B, Kunz U, Mathieu R, Kunz U, Mauer UM. Treatment of unresectable skull base meningiomas with somatostatin analogs. Neurosurg Focus 2011;30:E11. https://doi.org/10.3171/2011.1.FOCUS111.

[12] Simó M, Argyriou AA, Macià M, Plans G, Majós C, Vidal N, et al. Recurrent high ‑ grade meningioma : a phase II trial with somatostatin analogue therapy 2014:919–23. https://doi.org/10.1007/s00280-014-2422-z.

[13] Minutoli F, Amato E, Sindoni A, Cardile D, Conti A, Herberg A, et al. Peptide receptor radionuclide therapy in patients with inoperable meningiomas: our experience and review of the literature. Cancer Biother Radiopharm 2014;29:193–9. https://doi.org/10.1089/cbr.2013.1599.

[14] Marincek N, Radojewski P, Dumont RA, Brunner P, Muller-Brand J, Maecke HR, et al. Somatostatin Receptor-Targeted Radiopeptide Therapy with 90Y-DOTATOC and 177Lu-DOTATOC in Progressive Meningioma: Long-Term Results of a Phase II Clinical Trial. Journal of Nuclear Medicine 2015;56:171–6. https://doi.org/10.2967/jnumed.114.147256.

[15] Gerster-Gillieron K, Forrer F, Maecke H, Mueller-Brand J, Merlo A, Cordier D. 90Y-DOTATOC as a Therapeutic Option for Complex Recurrent or Progressive Meningiomas. Journal of Nuclear Medicine 2015;56:1748–51. https://doi.org/10.2967/jnumed.115.155853.

[16] Seystahl K, Stoecklein V, Schuller U, Rushing E, Nicolas G, Schafer N, et al. Somatostatin receptor-targeted radionuclide therapy for progressive meningioma: benefit linked to 68Ga-DOTATATE/-TOC uptake. Neuro Oncol 2016;18:1538–47. https://doi.org/10.1093/neuonc/now060.

[17] Hartrampf PE, Hänscheid H, Kertels O, Schirbel A, Kreissl MC, Flentje M, et al. Long-term results of multimodal peptide receptor radionuclide therapy and fractionated external beam radiotherapy for treatment of advanced symptomatic meningioma. Clin Transl Radiat Oncol 2020. https://doi.org/10.1016/j.ctro.2020.03.002.

[18] Müther M, Roll W, Brokinkel B, Zinnhardt B, Sporns PB, Seifert R, et al. Response assessment of somatostatin receptor targeted radioligand therapies for progressive intracranial meningioma. NuklearMedizin 2020. https://doi.org/10.1055/a-1200-0989.

[19] Lou E, Sumrall AL, Turner S, Peters KB, Desjardins A, Vredenburgh JJ, et al. Bevacizumab therapy for adults with recurrent/progressive meningioma: a  retrospective series. J Neurooncol 2012;109:63–70. https://doi.org/10.1007/s11060-012-0861-0.

[20] Nayak L, Iwamoto FM, Rudnick JD, Norden AD, Lee EQ, Drappatz J, et al. Atypical and anaplastic meningiomas treated with bevacizumab. J Neurooncol 2012;109:187–93. https://doi.org/10.1007/s11060-012-0886-4.

[21] Furuse M, Nonoguchi N, Kawabata S, Miyata T, Toho T, Kuroiwa T, et al. Intratumoral and peritumoral post-irradiation changes, but not viable tumor  tissue, may respond to bevacizumab in previously irradiated meningiomas. Radiat Oncol 2015;10:156. https://doi.org/10.1186/s13014-015-0446-0.

[22] Shih KC, Chowdhary S, Rosenblatt P, Weir AB, Shepard GC, Williams JT, et al. A phase II trial of bevacizumab and everolimus as treatment for patients with refractory, progressive intracranial meningioma. J Neurooncol 2016;129:281–8. https://doi.org/10.1007/s11060-016-2172-3.

[23] Kumthekar P, Grimm SA, Aleman RT, Chamberlain MC, Schiff D, Wen PY, et al. A multi-institutional phase II trial of bevacizumab for recurrent and refractory  meningioma. Neurooncol Adv 2022;4:vdac123. https://doi.org/10.1093/noajnl/vdac123.

[24] Alexander AY, Onyedimma C, Bhandarkar AR, Yolcu YU, Michalopoulos GD, Bydon M, et al. The role of bevacizumab for treatment-refractory intracranial meningiomas: a single institution’s experience and a systematic review of the literature. Acta Neurochir (Wien) 2022;164:3011–23. https://doi.org/10.1007/S00701-022-05348-X.

[25] Raizer JJ, Abrey LE, Lassman AB, Chang SM, Lamborn KR, Kuhn JG, et al. A phase I trial of erlotinib in patients with nonprogressive glioblastoma  multiforme postradiation therapy, and recurrent malignant gliomas and meningiomas. Neuro Oncol 2010;12:87–94. https://doi.org/10.1093/neuonc/nop017.

[26] Kaley TJ, Wen P, Schiff D, Ligon K, Haidar S, Karimi S, et al. Phase II trial of sunitinib for recurrent and progressive atypical and anaplastic meningioma. Neuro Oncol 2015;17:116–21. https://doi.org/10.1093/neuonc/nou148.

[27] Reardon DA, Desjardins A, Vredenburgh JJ, Herndon JE, Coan A, Gururangan S, et al. Phase II study of Gleevec plus hydroxyurea in adults with progressive or recurrent low-grade glioma. Cancer 2012;118:4759–67. https://doi.org/10.1002/cncr.26541.

[28] Ji Y, Rankin C, Grunberg S, Sherrod AE, Ahmadi J, Townsend JJ, et al. Double-Blind Phase III Randomized Trial of the Antiprogestin Agent Mifepristone  in the Treatment of Unresectable Meningioma: SWOG S9005. J Clin Oncol 2015;33:4093–8. https://doi.org/10.1200/JCO.2015.61.6490.

[29] Belanger K, Ung TH, Damek D, Lillehei KO, Ormond DR. Concomitant Temozolomide plus radiotherapy for high-grade and recurrent  meningioma: a retrospective chart review. BMC Cancer 2022;22:367. https://doi.org/10.1186/s12885-022-09340-7.

[30] Brastianos PK, Kim AE, Giobbie-Hurder A, Lee EQ, Wang N, Eichler AF, et al. Phase 2 study of pembrolizumab in patients with recurrent and residual high-grade meningiomas. Nat Commun 2022;13. https://doi.org/10.1038/S41467-022-29052-7.

[31] Yust-Katz S, Amiel A, Siegal T, Limon D. A phase II, open-label, single-arm trial of pembrolizumab for refractory atypical and anaplastic meningioma and hemangiopericytoma. Journal of Clinical Oncology 2021;39:2064. https://doi.org/10.1200/JCO.2021.39.15\_suppl.2064.

[32] Bi WL, Nayak L, Meredith DM, Driver J, Du Z, Hoffman S, et al. Activity of PD-1 blockade with nivolumab among patients with recurrent atypical/anaplastic meningioma: phase II trial results. Neuro Oncol 2022;24:101–13. https://doi.org/10.1093/NEUONC/NOAB118.

[33] Preusser M, Silvani A, Le Rhun E, Soffietti R, Lombardi G, Sepulveda JM, et al. Trabectedin for recurrent WHO grade 2 or 3 meningioma: A randomized phase II study of the EORTC Brain Tumor Group (EORTC-1320-BTG). Neuro Oncol 2022;24:755–67. https://doi.org/10.1093/NEUONC/NOAB243.

[34] Bi WL, Nayak L, Meredith DM, Driver J, Du Z, Hoffman S, et al. Activity of PD-1 blockade with nivolumab among patients with recurrent atypical/anaplastic meningioma: phase II trial results. Neuro Oncol 2022;24:101. https://doi.org/10.1093/NEUONC/NOAB118.

[35] Brastianos PK, Kim AE, Giobbie-Hurder A, Lee EQ, Wang N, Eichler AF, et al. Phase 2 study of pembrolizumab in patients with recurrent and residual high-grade meningiomas. Nat Commun 2022;13. https://doi.org/10.1038/S41467-022-29052-7.
