# Supplementary material for: The concept, intention, and evaluation of the term treatment-refractory meningioma
Source: J Neurooncol. 2025 Aug 4;175(2):599–610. doi: 10.1007/s11060-025-05154-2 (PMC12420741; doi:10.1007/s11060-025-05154-2)
Supplement: Supplementary file 2 — Supplementary Material 2 [file 11060_2025_5154_MOESM2_ESM.docx]

**Supplementary Table 3**. Ongoing clinical trials for treatment-refractory meningiomas, according to ClinicalTrials.gov

| **Investigator,**  **NCT number** | **Intervention** | **Mechanism of action** | **Cohort, enrollment** | **WHO grade** | **Definition of treatment-refractory/inclusion criteria** | **Reference group** |
| --- | --- | --- | --- | --- | --- | --- |
| Limon, NCT03016091 | Pembrolizumab | PD-1 inhibitor | Estimated 25 | Includes 2 and 3 | Histologically, previously proven, grade II or III meningioma, HPC or classic radiographic features of a recurrent surgically inaccessible atypical or anaplastic meningioma. All patients would have to have recurrence despite radiotherapy, unless radiotherapy is contraindicated. No limit on the number of prior surgeries, radiation or radiosurgery treatments. No limit on prior systemic therapies - chemotherapy or biological agents. Patients who received stereotactic radiosurgery (SRS) are eligible without histologic documentation of recurrence if at least 6 months have passed from previous SRS treatment, and preferably, but not necessarily a 2-ﬂuoro-2-deoxy-D-glucose PET imaging demonstrated hypermetabolism. | No |
| Brastianos, NCT03279692 | Pembrolizumab | PD-1 inhibitor | Actual 26 | Includes 2 and 3 | Histologically proven recurrent or residual intracranial or metastatic meningioma or meningioma with extracranial spread Progressive OR residual disease, as defined by the following: Progressive disease, as defined as an increase in size of the measurable primary lesion on imaging by 25% or more (bidirectional area). The change must occur between scans separated by no more than 24 months.  Residual measurable disease: For Grade II or III meningioma, residual measurable disease immediately after surgery without requirement for progression. Residual measurable disease will be defined by bidimensionally measurable lesions with clearly defined margins by MRI scans, with a minimum diameter of 10mm in both dimensions. Post radiation patients: Patients with measurable and progressive meningioma who have received radiation are potentially eligible, but need to show evidence of progressive disease in the radiated field after completion of radiation. At least 24 weeks must have elapsed from completion of radiation to registration. Patients that have progressive disease outside of the radiation field do not need to wait 24 weeks from completion of radiation.  No molecular classification. | No |
| Bush, NCT04659811 | Pembrolizumab + Stereotactic Radiosurgery | PD-1 inhibitor | Estimated 90 | Includes 1, 2 and 3 | For Stratum A, patients must have histologically confirmed World Health Organization (WHO) grade II or III meningioma that is progressive or with one or more recurrences following surgical resection and radiotherapy For Stratum B, patients must have histologically WHO-grade I meningioma WHO-are multiply recurrent (>= 2 recurrences) following surgical and radiotherapy intervention. Prior therapy: There is no limit on the number of prior surgeries or systemically administered therapeutic agents. For prior radiotherapy, there are no exclusions on number of courses or prior type of radiotherapy. All modalities including prior fractionated external beam photon/proton radiotherapy, stereotactic radiosurgery, and/or or brachytherapy are permissible with a required interval of 6 months from last prior radiotherapy treatment, unless radiation given outside of the planned field, then within 2 weeks from prior radiotherapy treatment. Patients must have had one prior course of radiation therapy. | No |
| Huang, NCT03604978 | Ipilimumab with/without Nivolumab and Stereotactic Radiosurgery | PD-1 inhibitor | Estimated 15 | Includes 2 and 3 | Patients must have histologically confirmed World Health Organization (WHO) grade II-III meningioma which has relapsed after prior radiation therapy with radiologically progressive or recurrent disease. Patients must have measurable disease, defined as at least 1 lesion that can be accurately measured in at least one dimension as >= 1 cm on brain magnetic resonance imaging (MRI) but with the maximum dimension =< 5 cm OR gross tumor volume < 20 cm^3. All the relapsed disease would need to be eligible to be treated with reirradiation. Patients must have at least one prior surgery with available archival formalin-fixed paraffin-embedded (FFPE) tumor blocks of the initial or recurrent meningioma. Prior initial radiation therapy may include external beam radiation or radiosurgery, or combination of both. However, the total dose of prior radiation exposure to the site of recurrent tumor (for consideration of re-irradiation) cannot be more than 70 Gy. The duration since the previous radiation exposure to the site of reirradiation need to be at least 6 months | No |
| Chen, NCT04728568 | Sintilimab | PD-1 inhibitor | Estimated 15 | Includes 3 | Pathologically diagnosed as a patient with WHO-grade III recurrent meningioma. Able to accept second surgery | No |
| Penas-Prado, NCT03173950 | Nivolumab | PD-1 inhibitor | Actual 108 (several tumor types) | Includes 2 and 3 | All types of CNS tumors.  For meningioma, histopathologically proven diagnosis Malignant/Atypical Meningioma. Patients with extra CNS metastases from meningioma will be eligible even if pathology review fails to demonstrate high grade features on available tumor samples. The tumor tissue must be available to be sent for immunophenotyping by NCI Laboratory of Pathology. Participants must have progressive tumor growth after having received established standard of care and/or other experimental treatments for their newly diagnosed or recurrent disease.  No molecular classification. | No |
| Reardon, NCT02648997 | Nivolumab and Ipilimumab | Nivolumab: PD-1 inhibitor  Ipilimumab: CTLA-4 inhibitor | Actual 40 | Includes 1, 2 and 3 | Have histologically confirmed WHO grade I, II or III meningioma that is progressive or recurrent. Metastatic meningiomas are allowed. Participants with grade I tumors must have failed radiation therapy. Prior therapy: There is no limit on the number of prior surgeries, radiation therapy, radiosurgery treatments or systemically administered therapeutic agents.  No molecular classification. | No |
| Huang, NCT03267836 | Avelumab  and Hypofractionated Proton Radiation | PD-L1 inhibitor and proton therapy | Actual 9 | Includes 1, 2 and 3 | Diagnosis of recurrent or progressive histologically confirmed WHO-grade I-III meningioma which has failed maximal safe resection and radiation therapy. At least one prior surgery with available archival formalin-fixed paraffin-embedded (FFPE) tumor blocks. In the case that tumor block is unavailable, unstained tissue sections may be used in its place. Prior treatment must include external beam radiation, radiosurgery, or combination of both. Deemed eligible for additional partial resection by treating physician and determined to be safe to receive 3 months of neoadjuvant therapy before planned surgery. | No |
| Sanai,  NCT02933736 | Ribociclib | CDK4/6 inhibitor | Estimated 48 (both glioma and meningioma) | Includes 2 and 3 | One prior resection of histologically-diagnosed World Health Organization (WHO) Grade III or IV glioma, or WHO grade II or III meningioma. For meningiomas, archival tissue much demonstrate (a) RB positivity on immunohistochemistry OR no RB mutations on next-gen sequencing (NGS). | No |
| Kaley,  NCT03220646 | Abemaciclib | CDK4/6 inhibitor | Actual 64 (several tumor types) | No info | Histologically confirmed IDH mutant glioma, meningioma, schwanomma, PCNSL, ependymoma, or other Primary Brain Tumors that have recurred despite previous standard of care therapy. Because this cohort is, in part, meant to allow patients access to therapy who might not otherwise be eligible for other clinical trials - deviations from standard of care treatment or histological confirmation can be presented to and approved by the Principal Investigator for inclusion in the study.  No molecular classification. | No |
| Sanai,  NCT05940493 | Abemaciclib | CDK4/6 inhibitor | Estimated 72 | Includes 3 | Participants who consent to the trial will have surgical tissue collected from the planned surgical resection and tested. If the tissue shows positive results for RB cells and participants are qualified, they will be enrolled and receive study treatment two to five weeks after completing standard-of-care radiation therapy.  Participant with an intracranial WHO Grade 3 meningioma or lower grade meningioma that has progressed to WHO grade 3 that have received prior radiation therapy.  Resected tissue must demonstrate: (a) RB positivity on immunohistochemistry (IHC); or, no RB mutations on next-generation sequencing (NGS). | Yes, placebo |
| Cordier, NCT04997317 | 177Lu-satoreotide | Somatostatin receptor–targeted radiopeptide | Estimated 18 | Includes 1, 2 and 3 | The participants must be patients with a histologically or clinically confirmed (MRI + somatostatin receptor imaging) recurrent or progressive meningioma. There must be no other standard therapeutic alternatives for the participants. | No |
| Sulman,  NCT03971461 | 177Lu-DOTATATE | Somatostatin receptor–targeted radiopeptide | Estimated 32 | Includes 1, 2 and 3 | Histologically confirmed diagnosis WHO-grade I-III meningioma: a. For grade I meningioma, subjects must have: i. Progressive disease after at least surgical resection and radiotherapy, as defined as an increase in size of the measurable primary lesion (bidirectional area) on imaging by 25% or more between scans separated by no more than 12 months. or ii. Progressive residual tumor after maximal safe resection, be located at or near critical organs at-risk and considered to be high-risk for radiation injury by the treating investigator. Prior external beam radiotherapy is not required for these subjects. b. For Grade II or III meningioma, subjects must have either: i. Progressive disease after at least surgical resection and radiotherapy, as defined as an increase in size of the measurable primary lesion (bi-directional area) on imaging by 25% or more between scans separated by no more than 12 months or ii. Residual measurable disease after surgery without requirement of progression. Multifocal disease is allowed but is limited to ≤ 3 measurable intracranial mass lesions on the most recent post-contrast MRI. | No |
| Merrell,  NCT04082520 | 177Lu-DOTATATE | Somatostatin receptor–targeted radiopeptide | Estimated 41 | Includes 1, 2 and 3 | Previous treatment for meningioma including surgery, when possible, and radiation therapy (conventional fractionated or radiosurgery). Pathologic confirmation of meningioma is not required for patients WHO-are not surgical candidates and received radiation therapy based on magnetic resonance imaging (MRI) consistent with meningioma. Patients with prior surgery will have pathologic confirmation of meningioma with either formalin-fixed paraffin-embedded (FFPE) tumor block OR meningioma tissue slides available for submission to central pathology review. Radiographic evidence of meningioma progression with measurable disease, defined as an increase in size of the measurable primary lesion on imaging by 15% or more (sum of the bidirectional measurements) in an approximate 6 month time period (i.e., calculated rate of growth 15% / 6 months based on available scans) or by the appearance of a new measurable lesion. Previous treatment with either fractionated radiation therapy or stereotactic radiosurgery at the site of progressive meningioma, without safe option for further radiotherapy. | No |
| Lazow,  NCT05278208 | 177Lu-DOTATATE | Somatostatin receptor–targeted radiopeptide | Estimated 65 | Includes 1, 2 and 3 | Diagnosis Patient must have a diagnosis of primary high-grade CNS tumor (any histopathologic diagnosis that is WHO-grade III-IV) or meningioma (any histologic grade) that is recurrent, progressive, or refractory. Note that patients with DIPG (based on radiographic/clinical diagnosis) WHO-have undergone biopsy will be eligible with histologic diagnosis of grade II-IV infiltrating glioma. All tumors must have histologic verification either at the time of diagnosis or recurrence, except for patients meningioma WHO-have not previously undergone biopsy or resection. Refractory disease is defined as the presence of persistent abnormality on conventional MRI imaging that is further distinguished by histology (biopsy or sample of lesion) or advanced imaging, OR as determined by the treating physician and discussed with the primary investigator prior to enrollment. Patients with meningioma WHO-have pre-trial tumor tissue available are required to submit tissue; however, this is not required for eligibility for meningioma patients if no prior biopsy/resection has been performed. Prior Therapy Patients must have recurred/progressed following prior standard therapy for their tumor. Note: with meningioma, atypical meningioma, or anaplastic meningioma must have received at least surgical resection or radiation. | No |
| EORTC*, NCT06326190 | 177Lu-DOTATATE | Somatostatin receptor–targeted radiopeptide | Estimated 135 | Includes 1, 2 and 3 | Histologically confirmed diagnosis of meningioma (all grades, 1-3 per WHO CNS5, are eligible). At least one prior surgery and one line of external beam radiotherapy for meningioma. Exclusion Criteria: Local therapy (surgery and / or radiotherapy) indicated per local investigator. Note: in case of patients with multiple meningioma lesions, in whom resection and / or radiotherapy of individual lesions is indicated, patients may be included after local therapy (with a 4-week gap between surgery / end of radiotherapy and start of treatment), if at least one remaining lesion fulfils the inclusion criteria. Any combined or any prior systemic treatment regardless the timing. | Control group: local standard of care According to local standard practice, treatment in the control arm is left to the investigator's discretion. |
| Verger, NCT06126588 | 177Lu-DOTATATE and Everolimus | Somatostatin receptor–targeted radiopeptide + Anti-VEGF antibody | Estimated 28 | Includes 2 and 3 | Patient with grade 2 and 3 meningioma, substantiated by histology, not amenable to surgery or radiotherapy, with clinical or radiological progression. Clinical deterioration or at least 10% of tumor growth rate, defined as the product of the two largest diameters of the target lesion within 6 months. | No |
| Boursier, NCT06255249 | 177LUTEtium-oxodotreotide | Somatostatin receptor–targeted radiopeptide | Estimated 50 | Includes 1, 2 and 3 | Patients who have benefited from treatment with Lutathera within the framework of compassionate prescription in a refractory meningioma of any grade | No |
| Schembri, NCT03936426 | Cu-64 SARTATE and Cu-67 SARTATE | Somatostatin receptor–targeted radiopeptide | Actual 5 | Includes 1, 2 and 3 | Diagnosis of recurrent or progressive histologically confirmed WHO-grade I-III meningioma which has failed standard of care therapies. Patients will be considered to have failed standard care when they have disease that is progressing despite standard treatment (primarily radiotherapy) or where, in the opinion of their treating physician, further standard therapy is considered to be of sufficiently high risk of complication as to warrant consideration of alternate therapies. | No |
| Wen, NCT00859040 | Pasireotide (SOM230C) | Somatostatin analogue | Actual 34 | Includes 1, 2 and 3 | Histologically confirmed diagnosis of recurrent or progressive intracranial meningioma(s). This includes benign, atypical, or malignant meningioma; patients with neurofibromatosis type 1 or 2 may participate. Participants without histological confirmation but a classic radiographic picture of meningioma may also enroll. Patients with neurofibromatosis type 2 and a classic radiographic picture of meningioma may also enroll without histological confirmation. There is no limit on the number of prior therapies. | No |
| Plotkin, NCT03071874 | Vistusertib | Dual mTORC1/mTORC2 Inhibitor | Actual 28 | Includes 2 and 3 | Participants must have histologically confirmed intracranial meningioma, grade II-III,that has recurred or progressed at previous treatment. Patients must have received prior surgical resection and radiation therapy for the progressive meningioma. Patients must have received less than three prior chemotherapy regimens for progressive meningioma. | No |
| Hainsworth, NCT00972335 | Bevacizumab and Everolimus | Anti-VEGF antibody + mTOR inhibitor | Actual 18 | Includes 1, 2 and 3 | Histologic diagnosis of meningioma, WHO-grade 1, 2, or 3 (benign, atypical, or malignant). In addition, patients with definitive radiologic evidence of meningioma WHO-are unresectable, and in whom radiation therapy without biopsy is the standard treatment, are also eligible. All patients must have developed recurrent disease/progression after receiving all standard treatments, which must include the following: surgical resection, if possible; definitive radiation therapy for unresectable meningioma, or for recurrent meningioma after resection. patients must be at least 4 weeks post-surgery, and must be at least 2 weeks post-radiation therapy, with resolution of related toxicities. All patients must have progressive symptoms judged to be directly related to their recurrent/progressive meningioma. Patients with no new symptoms, or patients with stable neurologic deficits from previous surgical resection, are not eligible. Patients may have had 0 or 1 previous systemic treatment regimens. | No |
| Graillon, NCT02333565 | Everolimus and Octreotide | Anti-VEGF antibody + Somatostatin analogue | Completed. 20 | Includes 1, 2 and 3 | Histologically proven meningioma grade II and III; grade I meningioma may also be included, if progression is documented (see criteria 3), particularly in case of skull base location. Patients must have failed surgery, and not amenable to a new curative intended surgery Patients must have failed radiotherapy and/or radiosurgery Prior chemotherapy is allowed, if progression under the cytotoxic agent is clearly documented. An interval of 4 weeks after the last administration of the cytotoxic agent is warranted. Number of prior chemotherapies is not limited. | No |
| Kumthekar,  NCT02847559 | Bevacizumab | Anti-VEGF antibody | Estimated 27 | Includes 2 and 3 | Patients must have a histologic diagnosis of meningioma, World Health Organization (WHO) grade 2 or 3 (atypical or anaplastic). Patient's tumor must have a supratentorial component Patients must have measurable or non-measurable (evaluable) disease recurrence; recurrence must be documented by magnetic resonance imaging (MRI) or computed tomography (CT) scan. All patients must have developed recurrent disease/progression (evidence of recurrence to be established by MRI or CT scan with contrast; there is no limit to the number of relapses) after receiving all standard treatments, which must include the following: Surgical resection, if possible; Definitive radiation therapy for unresectable meningioma, or for recurrent meningioma after resection. Patients may have had previous systemic treatment regimens with the exception of bevacizumab (no limit to number of prior therapies); a 4 week wash-out period prior to registration is mandatory for all systemic treatments | No |
| Zhang, NCT04501705 | Apatinib | Anti-VEGF antibody | Estimated 29 | Includes 2 and 3 | The pathological diagnosis of atypical/malignant meningioma was clear after biopsy or surgery. The tumor recurrence is confirmed by MRI, that is, the diameter of the lesion on the enhanced MRI image is ≥1cm, and ≥2 slices (slice interval 5mm) are visible; or after another biopsy or surgery, the pathological diagnosis is atypical/malignant meningioma. Previous surgery and radiotherapy (including conventional radiotherapy or stereotactic radiosurgery treatment) are required. There are no restrictions on whether to receive chemotherapy or the number of times of the above treatments | No |
| De Salvo, NCT06275919 | Regorafenib | Tyrosine kinases and VEGFR inhibitor, and PDGFRB and FGFR1 inhibitor | Estimated 104 | Includes 2 and 3 | Histological diagnosis of grade 2 or grade 3 meningioma according to the WHO 2021 classification. Ineligible for further surgery and/or radiotherapy. Radiologically documented progression (estimated planar growth >15%- measured in two-dimensional tumor area- within the prior 6 months or a new lesion develops) | Active comparator: Drug: Local Standard of Care. Local Standard of Care until disease progression or unacceptable toxicity |
| Kotecha,  NCT05425004 | Cabozantinib | Tyrosine kinases c-Met and VEGFR-2 inhibitor | Estimated 24 | Includes 1, 2 and 3 | Histologic (preferred) or radiologic diagnosis of meningioma. All WHO-grades (I, II and III) are allowed. All patients must have developed recurrent disease or progressive disease after receiving standard therapy (eg, radiation or surgery) >6 months ago or have been deemed ineligible to receive these therapies. | No |
| Graillon,  NCT03631953 | Alpelisib + Trametinib | Pi3Kα specific inhibitor and MEK inhibitor | Estimated 25 | Includes 1, 2 and 3 | Histologically proven meningioma grade I, II and III. Progression is defined as growing meningiomas on 2 different Magnetic Resonance Imaging (MRI) 3 to 6 months apart. Patients must have failed surgery, and not amenable to a new curative intended surgery .Patients must have failed radiotherapy and/or radiosurgery | No |
| Chen, NCT05023018 | NEO100 | Perillyl alcohol | Estimated 30 | Includes 2 and 3 | Have histologically confirmed WHO-Grade II or III meningioma that is residual, progressive or recurrent following at least minimally safe resection and radiation therapy. Metastatic meningiomas are allowed. Be on a stable or decreasing dose of steroids for at least five days prior to the date of informed consent. Participants must have failed maximal safe resection and radiation therapy. There is no limit on the number of prior surgeries, radiation therapy, radiosurgery treatments or systemically administered therapeutic agents. Patients' tumor must not be >30 mm (length x width) and must not be multifocal Participants must have recovered to grade ≤1 or pretreatment baseline from clinically significant adverse events related to prior therapy (exclusions include, but are not limited to alopecia, laboratory values listed per inclusion criteria and lymphopenia). | No |
| Rogers, NCT06012929 | ONC201 (dordaviprone) | Dopamine receptor antagonist and allosteric agonist of the mitochondrial protease caseinolytic mitochondrial matrix peptidase proteolytic subunit (ClpP) | Estimated 27 | No info | Patients who have previously received treatment for their meningioma but have exhausted all reasonable treatment options will be enrolled. Pathologically proven meningioma without reasonable surgical options for complete resection, or reasonable radiation therapy options, determined by neurosurgery and radiation oncology opinions. Any number of prior medical therapies is allowed but not required. Multifocal disease is allowed. | No |
| Brastianos,  NCT02523014 | Vismodegib,  FAK Inhibitor GSK2256098, Capivasertib, Abemaciclib | Vismodegib: Inhibits the Hedgehog signaling pathway by targeting Smoothened (SMO)  FAK inhibitor (GSK2256098): Blocks focal adhesion kinase (FAK)  Capivasertib: Inhibits AKT (protein kinase B), blocking PI3K/AKT signaling  Abemaciclib: CDK4/6 inhibitor | Estimated 124 | No info | Molecular documentation: Presence of SMO, PTCH1, NF2, CDKN2A, AKT1, PIK3CA, PTEN mutations, CDKN2A copy number loss, CDK4, CDK6, CCND1, CCND2, CCND3, or CCNE1 copy number gain in tumor sample as documented specifically by the central laboratory.  Progressive OR residual disease, as defined by the following: Residual measurable disease: residual measurable disease immediately after surgery without requirement for progression; for grade I disease, progression pre-operatively needs to be documented, with an increase in size of the measurable primary lesion on imaging by 25% or more (bidirectional area); the change must occur between scans separated by no more than 25 months; for patients with SMO/PTCH1 mutations enrolling to receive vismodegib, the change can occur between scans separated by up to 25 months; residual measurable disease will be defined by bidimensionally measurable lesions with clearly defined margins by MRI scans, with a minimum diameter of 10 mm in both dimensions.  Progressive measurable disease: progression defined as an increase in size of the measurable primary lesion on imaging by 25% or more (bidirectional area); the change must occur between scans separated by no more than 25 months.  Post radiation patients: patients with measurable and progressive meningioma who have received radiation are potentially eligible, but need to show evidence of progressive disease after completion of radiation; if the progressive meningioma lesion has been radiated, at least 24 weeks must have elapsed from completion of radiation to registration; if the progressive lesion is outside of the radiation field, then an interval of at least 2 weeks must have elapsed from completion of radiation to registration. | No |
| Plotkin, NCT04374305 | Brigatinib  or  Neratinib | Brigatinib: ALK inhibitor  Neratinib: Irreversible tyrosine kinase inhibitor (TKI) targeting HER2 and EGFR | Estimated 100 (several tumor types) | No info | Patients must have a pathogenic variant in the NF2 gene (either in the germline or in two NF2-related tumors) OR a confirmed diagnosis of NF2 by fulfilling National Institute of Health (NIH) criteria or Manchester criteria. Participant must have a target NF2-related tumor with the following qualities: Not amenable to surgery due to patient refusal or due to high risk for surgical complications (e.g., damage to nerve function).  No molecular classification. | No |
| Recursion Pharmaceuticals Inc.,  NCT05130866 | REC-2282 | Histone deacetylase (HDAC) inhibitor | Estimated 92 (several tumor types) | No info | Progressive meningioma that is amenable to volumetric analysis. Has either 1) sporadic meningioma with confirmed NF2 mutation; or, 2) confirmed diagnosis of NF2 disease (revised Manchester criteria); or, 3) at least one NF2-related tumor (with pathogenic germline or proven mosaic NF2 variant).  Exclusion Criteria: (1) Progressive disease associated with significant or disabling clinical symptoms likely to require surgery or radiation therapy within the next 3 months. (2) Received prior surgery, radiosurgery, or laser interstitial thermal therapy in the target tumor, or immediately adjacent to the target tumor within 6 months prior to screening.  No molecular classification. | No |
| Kim, NCT05228015 | IK-930 | TEAD (Transcriptional Enhanced Associate Domain) inhibitor | Actual 67 (several tumor types) | No info | Subjects with histologically proven advanced, unresectable, locally recurrent, or metastatic malignancy that has progressed on or following standard-of-care therapies and for whom there is no available therapy known to confer clinical benefit, regardless of the presence or absence of NF2 deficiency or other genetic alterations of the Hippo pathway. Subjects with histological confirmation of MPM; subjects with NF2-deficient MPM determined by local test results for testing can also be enrolled as well as subjects with any other solid tumors with documented NF2 deficiency determined by local test results for testing, including, but not limited to, meningioma, cholangiocarcinoma, thymoma, mucoepidermoid NSCLC, HCC, and others. Subjects diagnosed with EHE with documented TAZ-CAMTA1 or YAP1-TFE3 gene fusions, as determined by local tests and subjects with solid tumors who have YAP1/TAZ gene fusions as determined by local test results can also be enrolled in the dose escalation part of the study. | No |

*European Organization for Research and Treatment of Cancer
